# Supplementary material for: Patterns of Intron Gain and Loss in Fungi
Source: PLoS Biol. 2004 Nov 30;2(12):e422. doi: 10.1371/journal.pbio.0020422 (PMC532390; doi:10.1371/journal.pbio.0020422)
Supplement: Table S1 — Also available at http://genes.mit.edu/NielsenEtAl/. (4.3 MB ZIP). [file pbio.0020422.st001.zip › NielsenEtAl/html/1414.html]

AN5181.1.NCU02588.1.MG03604.1.FG09165.1


```
 CLUSTAL W (1.82) Multiple Sequence Alignments - Introns Inserted


Sequence 1: NCU02588.1	191 aa
Sequence 2: FG09165.1	188 aa
Sequence 3: MG03604.1	185 aa
Sequence 4: AN5181.1	198 aa
Alignment Length: 201 aa
Number Identitical Residues: 92 aa
Alignment Score (without introns) 4644


MG03604.1 	MTDK-~-------~-----E1AAARAKEAAEQAALPYKWAQTIGDIDVTIKLGEGSEKYK
NCU02588.1	MADKD1VT----E~LTPAEE~AAARAKEQAEQEALPYKWTQTIGDVDITILVPG---NLK
FG09165.1 	MTDK-~------E1PTPAET~AAATAKEAEEQAALPYKWTQTISELNATFDVPG---NLK
AN5181.1  	MSEQE~PSSADLA~AREAEE~KQRKAAEEAEQATLPYKWTQTIRDVDVTIPVSA---NLK
          	*:::.  :::.      :.      * *  ** :*****:*** ::: *: :     : *

MG03604.1 	GKDLKVEIKKQHLTAGVKGS--DPIID0GDLPHAVRVDESTWTLTTNPDG-TKNLEVHLD
NCU02588.1	GKDMVVEIKKQSLTAGIKGQ--EPVIK0GDLPHPILLDDSTWTLSPAPAG-QKALEIHLE
FG09165.1 	SRDLVITIKKMSLQAGIKGQ--DPIIQ0GDLPHAVHVDDSTWTLSTNSDG-TKTVEIHLD
AN5181.1  	GRDLDVVLKKDSIKVKVKGENGEVFID0GQFPHPIKPSESSWTLETTSKPPGKEVSIHLD
          	.:*: : :**  : . :**...: .*. *::**.:  .:*:*** . .  . * :.:**:

MG03604.1 	KVNKMEWWPHVVTDAPKIDVTKINPENSKLSDLDGETRGMVEKMMYDQRQKELGKPSSDE
NCU02588.1	KHNKLEWWPHVVTSAPKIDVSKIVPENSKLSDLEGETRAMVEKMMYDQRQKEMGLPTSDE
FG09165.1 	KVNKMEWWPHVVTSAPKIDVTKIQPDNSKLSDLDGETRGMVEKMMFDQQQKEKGLPSSDE
AN5181.1  	KVNQMEWWAHVVTTAPKIDVSKITPENSSLSDLDGETRAMVEKMMYDQRQKEMGAPTSDE
          	* *::***.**** ******:** *:**.****:****.******:**:*** * *:***

MG03604.1 	QKKLDLLAKFQKEHPE~MDFSNAKIN
NCU02588.1	QKKMDILKKFQEQHPE~MDFSNAKIQ
FG09165.1 	QKKADILKKFQEQHPE~MDFSKAKIQ
AN5181.1  	QRKMDILKKFQKEHPE1MDFSNAKIG
          	*:* *:* ***::*** ****:***
```
